# The Effect of Community Exercise Interventions for People with MS Who Use Bilateral Support for Gait

**DOI:** 10.1155/2014/109142

**Published:** 2014-01-02

**Authors:** Neasa Hogan, Maria Kehoe, Aidan Larkin, Susan Coote

**Affiliations:** ^1^Clinical Therapies Department, University of Limerick, Limerick, Ireland; ^2^Multiple Sclerosis Ireland, Galway, Ireland

## Abstract

*Background*. Mobility limitations are a key feature of MS and 25% will require the use of a walking aid 15 years after diagnosis. Few studies have specifically evaluated the effectiveness of physiotherapy and exercise interventions delivered in the community for those with significant disability. *Methods*. An assessor blind, block randomised, and controlled study recruited participants who required bilateral assistance for gait and who occasionally used wheelchairs for longer distances. They were randomised to 10 weeks of group physiotherapy (balance and strengthening exercises), individual physiotherapy, yoga group, or a control group. *Results*. Repeated measures ANOVA found significant time effects for physical component of MSIS-29v2 (*f* = 7.993, *P* = 0.006) and MFIS (*f* = 8.695, *P* = 0.004). The group × time interaction was significant for the BBS (*f* = 4.391, *P* = 0.006). Post hoc analysis revealed no difference between group and individual physiotherapy for BBS. There was no significant difference between groups but the 6MWT improved for individual physiotherapy (*P* = 0.001) and MSIS-29v2 psychological score for group physiotherapy (*P* = 0.005). *Discussion*. This study found that balance and strengthening exercises, delivered in the community to those with significant mobility limitations, improve balance. The effect on walking endurance and patient-reported outcomes are unclear and warrants further investigation with a larger control group with similar baseline characteristics to the intervention groups.

## 1. Introduction

Multiple sclerosis is a chronic progressive disease that results in a number of symptoms including weakness, spasticity, tremor, ataxia, sensory disturbance, and balance disruption [1]. It is suggested that within 15–25 years of diagnosis approximately 50% of people with MS (PwMS) will require the use of a walking aid [2, 3]. Mobility loss is significantly associated with loss of employment and with increasing assistance with activities of daily living (ADLS) [4] and PwMS identify the continued loss of mobility as one of their greatest concerns for their future [5]. Loss of mobility and its resultant impact on every day activities are reflected by the increasing cost of care with greater disability [6].

There is an ever-expanding body of the literature to suggest the positive effect of exercise and physiotherapy interventions. Those who use walking aids have been included in several studies of physiotherapy interventions [7–10], progressive resistance training (PRT) [11, 12], aerobic exercise [13–15], and a combination of the above [16], and generally the studies have had positive outcomes. However, as the results of those who use walking aids have not been analysed separately, it is difficult to evaluate the effect of the interventions for this specific population. Given the combinations and severity of their symptoms and resulting activity limitations, it is possible that they will respond differently to those with minimal disability.

Few studies have specifically focused on those with significant walking limitations that require support for gait (EDSS 6-7). Recently Pilutti et al. [17] found that treadmill training with body weight support resulted in significant improvements in quality of life, with large effect sizes for fatigue. A survey of current practice in physiotherapy [18] found that most people with MS who use walking aids are treated at home or in community or primary care settings. There is therefore a need for effectiveness trials of exercise interventions in these settings. Two studies were found that specifically evaluated interventions at home for people with MS who used walking aids. A pilot study of FES cycling at home [19] found trends for improvement in strength, walking speed, and endurance and in those muscles that were treated with electrical stimulation but the results need to be confirmed in a larger sample. In contrast Miller et al. [20] found no significant effect of home-based therapy, which consisted of twice weekly physiotherapy for eight weeks. Their small sample size may have influenced findings, which found some increases in impact of MS and strength for the intervention group. There is therefore a need for studies specifically exploring the responses of people with MS who use bilateral support for gait to interventions provided in the community. In Ireland the MS society provides physiotherapy and yoga interventions in community settings and commissioned this study to investigate their effectiveness.

The objective of this study was to evaluate the effectiveness of yoga and physiotherapy interventions delivered in community settings for people with significant disability due to MS who use bilateral aids for gait. The aim of this study was to compare the effects of a ten-week programme of group physiotherapy, individual physiotherapy, and yoga in the community to a control group who were asked not to change their exercise habits. Given the complex interactions of symptoms of this group we hypothesised that individual physiotherapy intervention would have more positive outcomes than group intervention and that physiotherapy interventions would have a greater outcome than that due to yoga. We also hypothesised that treatment would either prevent deterioration or bring about improvement compared to the control group.

## 2. Materials and Methods

The protocol for this study has been published previously [21]. This paper concerns the results for “strand B” or those people with MS who score 3 or 4 on the mobility section of the Guys Neurological Disability Rating Scale [22]. This indicates that they use bilateral assistance for gait and may use a wheelchair for longer distances. The study received ethical approval from the relevant committees in the 10 regions where the study took place. The trial registration number is ISRCTN77610415.

### 2.1. Participants

People who had their MS diagnosis confirmed by a consultant physician were included in the study. People with MS were excluded from the study if they had experienced an exacerbation of symptoms due to relapse or initiated steroid treatment in the last 12 weeks, were pregnant at the time of referral, or were under 18 years of age.

### 2.2. Procedure and Randomisation

Referral was made to the regional offices of MS Ireland by the persons themselves, their physiotherapist, general practitioner, consultant, or nurse. Participants were screened for eligibility, sent the relevant information leaflet, and gave informed consent. Once consent for 8 people in that geographical region was obtained, they were block randomised to one of the three interventions or to the control group by the national coordinator in MS Ireland. The randomisation sequence was generated by removing strips of paper from an envelope and was concealed from all involved in the study until the point of randomisation of that group. It should be noted that a number of blocks of participants were not treated as randomised. Groups allocated to yoga and to the control group lobbied to receive physiotherapy and were subsequently randomised to either group or individual physiotherapy.

### 2.3. Assessments

Assessments were carried out at baseline (week 1) and postintervention (week 12) by a blinded assessor. The following outcome measures were used: Berg Balance Scale (BBS) six-minute walk test (6MWT), Multiple Sclerosis Impact Scale 29 version 2 (MSIS), and Modified Fatigue Impact Scale (MFIS).

The BBS is a clinical scale that evaluates balance in sitting and standing and rates performance from 0 (cannot perform) to 4 (normal performance). The BBS was found to have a good concurrent validity and a cut-off score of 44 (out of 56) was established as a criterion to identify PwMS who have high risk of falls [23]. It was found to have high test-retest and interrater reliability, both having intraclass correlation coefficients (ICCs) of 0.96 [23].

In the 6MWT, the participants were asked to walk for a period of six minutes and the distance walked was recorded. Paltamaa et al. [24] found that the 6MWT is highly reliable in people with mild-to-moderate MS (EDSS 2–6.5) and Marrie and Goldman [25] validated the 6MWT as an outcome measure for PwMS. Subjects were instructed to walk “as quickly and safely as possible” as recommended by Fry and Pfalzer [26].

The MSIS-29v2 is a patient-reported outcome measure that assesses the physical and psychological impact of MS. It consists of 29 questions and is scored from 0 to 100 with higher scores indicating a greater impact of MS. It was developed and evaluated using robust psychometric techniques and is valid, reliable, and sensitive to change in PwMS [27–29].

The MFIS is a structured self-report questionnaire. It consists of 20 questions and is scored from 0–84. A higher score indicates a greater impact of fatigue. The MFIS has good reproducibility in an international sample of people with MS [30]. A cutoff of 38 points indicates clinically relevant fatigue [31].

### 2.4. Interventions

All interventions took place for an hour a week, for 10 weeks. The physiotherapy group intervention was a self-paced circuit style class of exercises that targeted strength and balance with the aim of increasing balance and mobility. The exercises were adapted from the falls prevention literature where similar programmes have been seen to improve, balance, and reduce the number of falls in an elderly population [32] and in people with MS [11].

The six exercises and possible progressions are described in Table 1. These were performed in sets of 12 at a self-paced rate. When a participant was able to perform 12 repetitions of an exercise safely, it was progressed up to 3 sets of 12 repetitions. The progression was dependent on the ability of the participant and their safety while performing the exercises.

The participants allocated to individual physiotherapy received individual treatment depending on the problem list and goals established by the Chartered Physiotherapist who was treating them. The content of the intervention was recorded for each individual treatment session. The duration of the individual sessions was the same as the group led physiotherapy.

Participants attended a weekly yoga class of approximately one-hour duration. All yoga instructors were members of The Yoga Federation of Ireland and kept a log of the content of each yoga class.

### 2.5. Analysis

All data was analysed using Predictive Analytic Software (PASW) Statistics 17. The distribution of the data was analysed for normality using Histograms, Quantile-Quantile plots, and the Shapiro Wilk statistic. Baseline differences between groups were assessed using one-way ANOVA (normally distributed data), Kruskal Wallis (nonnormally distributed data), and Chi square test for independence (categorical data).

Repeated measures ANOVA was performed for the physical component of the MSIS-29v2, the MFIS, and the BBS due to the normal distribution of the data. Bonferroni corrections were made and the *P* values were adjusted accordingly by PASW. Post hoc analysis was conducted using paired-samples *t*-tests for within-group changes and independent *t*-tests on the change scores for between-group differences. For the psychological component of the MSIS-29v2 and the 6MWT the normality tests yielded *P* values of < 0.05 and the histograms revealed skewed data; therefore, the assumptions of parametric testing were not met. Wilcoxon Signed Rank Tests were used to analyse the differences within groups and the Kruskal Wallis and Mann Whitney *U* tests were used to analyse the differences in change scores between groups.

## 3. Results

**Figure 1:**
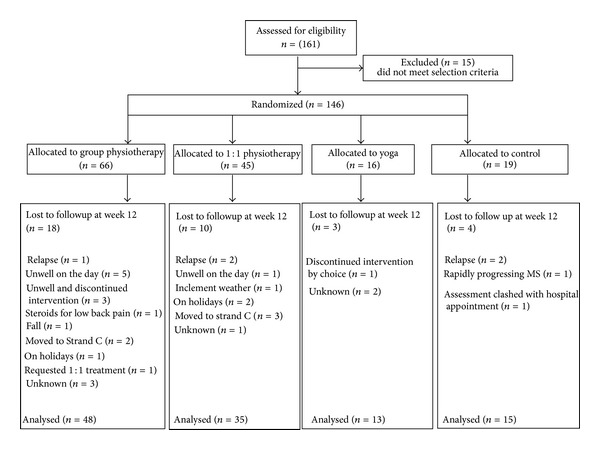
Flow of participants through the trial.

The flow of participants through the trial is illustrated in Figure 1. At baseline the control group were significantly younger and had a significantly shorter time since diagnosis. Also the yoga group had significantly less impact of fatigue (Table 2). The overall attrition rate for the study was 22.32%. The reasons for attrition and the rate of dropouts were similar across the groups (Figure 1).

The median number of sessions attended was 8 (semi-interquartile range 1.5), 9 (1), and 8 (2.25), for group physiotherapy individual physiotherapy and yoga, respectively. There was no significant difference between the three intervention groups for the number of sessions attended, *P* = 0.139 using the Kruskal-Wallis test. Analysis of the documentation of the content of the interventions revealed that group physiotherapy was performed as prescribed.

For individual physiotherapy, three out of the four physiotherapists delivered strength and balance exercises that were similar to those prescribed for the group physiotherapy in addition to other treatments specific to the individuals' problems. Additional components included pacing techniques, specific lower back exercises, walking, stretching, and bridging exercises. There were three yoga led groups. Relaxation, meditation, breathing techniques, and stretching were common to all three classes. One of the three classes included squatting, which was one of the prescribed exercises for the group physiotherapy intervention. Other components that made up the yoga classes included maintaining different static poses, for example, the mountain pose, the cat pose and the tailor pose (2 classes), and self-massage (1 class).

The descriptive statistics and post hoc tests for all outcome measures are presented in Table 3. Repeated Measures ANOVA showed a significant time effect for the physical component of the MSIS-29v2 (*f* = 7.993, *P* = 0.006) and the MFIS (*f* = 8.695, *P* = 0.004). The group × time interaction was significant for the BBS (*f* = 4.391, *P* = 0.006). All interventions showed a statistically significant improvement from Week 1 to Week 12 on the BBS which was greater than the control group. Post hoc analysis using an Independent *t*-test revealed there was no significant difference between Group and one to one physiotherapy on the BBS (*P* = 0.242). Due to the small numbers in the yoga group, post hoc comparisons to the physiotherapy groups were not conducted.

Nonparametric analysis revealed a significant change over time on the 6MWT for one-to-one physiotherapy (*P* = 0.001) and on the MSIS-29v2 psychological component for group physiotherapy (*P* = 0.005). The Kruskal Wallis test showed that there was no statistically significant difference between groups for the psychological component of the MSIS-29v2 or the 6MWT.

The control group showed a similar magnitude of change to the physiotherapy intervention groups on the self-report outcome measures.

**Figure 2:**
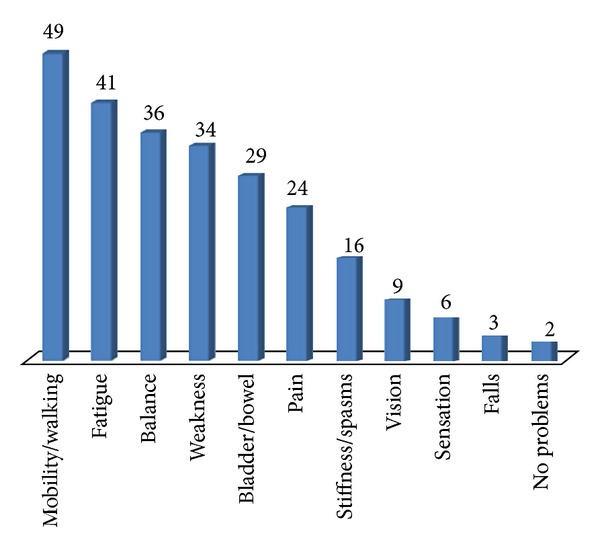
Main problems reported by participants.

The main problems reported by the participants are presented in Figure 2. Mobility, fatigue, and balance were the most commonly reported main problem.

## 4. Discussion

This is only one of a few trials that specifically address the effects of interventions in the community for those with significant mobility limitations who use bilateral support for gait. These data provide preliminary evidence of the effectiveness on balance of group and individual physiotherapy interventions delivered pragmatically in community settings.

The only evidence to support our hypotheses that treatment improves outcomes is from the BBS results. All three intervention groups improved significantly while the scores of the control groups worsened. The worsening of scores in the control group is not unexpected in this group who present primarily with progressive MS. The magnitude of the mean improvements in our study was similar to those of Cattaneo et al. [7] who found an improvement of 4.6 as a result of balance exercises based on motor strategies. However, this degree of change is less than the 6.5 value for the minimal detectable change for people with MS [33]. It should be noted, however, that our participants started with far lower BBS scores (22–30) than those evaluated in previous studies (>40); therefore, this degree of change may be clinically relevant to this degree of balance deficit. Interestingly this improvement in balance does translate to a reduction in both falls risk and number of falls [34]. Because of the small sample sizes confirmation of the effect of yoga is required and the effect of physiotherapy interventions needs further comparison to a larger, matched, and control group.

We hypothesised that individual physiotherapy would be more effective than the group interventions. While individual physiotherapy was tailored to the individual goals and impairments of the participants, the group intervention aimed to improve balance and mobility and was tailored only to ability level for those exercises. Post hoc analysis of the individual and group physiotherapy treatments suggests that there were no differences on the BBS between groups. As this trial was not planned as a noninferiority study, further comparison of group and individual physiotherapy for this population is warranted using a range of outcomes.

The main problem reported by the participants was walking/mobility limitations. The percentage improvements in 6MWT were 20.1% for group and 19.4% for individual physiotherapy, while the yoga group worsened by 35%. The high variability in the 6MWT data at baseline and in response to treatment may have led to nonsignificant findings despite relatively large median and % changes. The lack of deterioration in the control group is conflicting with the BBS data and suggests that further evaluation of this measure with larger groups with similar baseline characteristics is required.

There is conflicting evidence around the efficacy of yoga for mobility outcomes in PwMS in people with minimal gait impairment. Ahmadi et al. [35] evaluated the effect of yoga in those with less gait limitations and found small but statistically significant effects on 2MWT and 10MWT. The other strand of this study (those who use at most a stick to walk) did not have statistically significant improvements in 6MWT following yoga [36]. The lack of improvement in walking endurance in both arms of this study may reflect the specificity principles of exercise; they practiced static poses and not the specific elements and task of walking.

The finding that participants randomised to the yoga and control groups lobbied to receive immediate physiotherapy interventions suggests that this cohort perceive a need or preference for physiotherapy. Hence, these data provide some new information about the treatment preferences of those people with MS who require bilateral support for gait.

Patient preference may have contributed to the deterioration in self-reported measures in the yoga group, which conflicts with the objective measure of balance. A review of studies that used a randomised preference design [37] found that patients who received their preference had significantly greater improvements. Additionally, authors have suggested a “resentful demoralisation” [38] when patients do not receive their preferred treatment and this may explain the deterioration in the self-reported measures in the yoga group.

### 4.1. Limitations

There are a number of methodological limitations in this study. Selection bias arose as several groups were not treated as randomised. This resulted in detection bias as the groups were not similar at baseline. At baseline, the control group were significantly younger and had a shorter time since diagnosis. However, they were similar for all clinical measures with the exception of the MFIS score for the yoga group. The score of 30.4 on the MFIS for the yoga group is lower than the cutoff of 38 for clinically meaningful fatigue and may indicate that they were not significantly fatigued to start with.

An additional element of detection bias is present as it was not possible to “blind” the participants to their group allocation. This is not unique to this study and remains a challenge in rehabilitation research.

The contact with an assessor on two occasions may also have led to elements of performance bias. While not statistically significant the control group improved on the self-reported outcome measures to the same magnitude as the treatment groups. This suggests that there may be a “placebo” effect of contact on the impact of MS and impact of fatigue.

The dropout rate across the study was 22% and contributes to attrition bias. Completer analysis is presented as it is inappropriate to impute values for intention to treat analysis when more than 20% of the data is missing [39]. Given the variable nature of the disease it was also felt that other forms of intention to treat analysis were not appropriate as the rate of deterioration or improvement between participants is variable and unpredictable.

## 5. Conclusions

These data provide preliminary evidence that 10-week interventions consisting of balance and strengthening exercises improve balance; however, given the methodological limitations of the trial, confirmation of these findings with a larger, matched control group is required. Post hoc analysis of the data suggests that the response to group and individual physiotherapy for balance is similar but this also requires confirmation in a trial that aims to compare this effect directly. People with MS who use bilateral support for gait indicated their preference for physiotherapy interventions over waiting three months for treatment or participating in yoga. Patient preference and the placebo effect of contact may have influenced the patient-reported outcome measures.

## Figures and Tables

**Table 1:** Group physiotherapy exercises and their progressions.

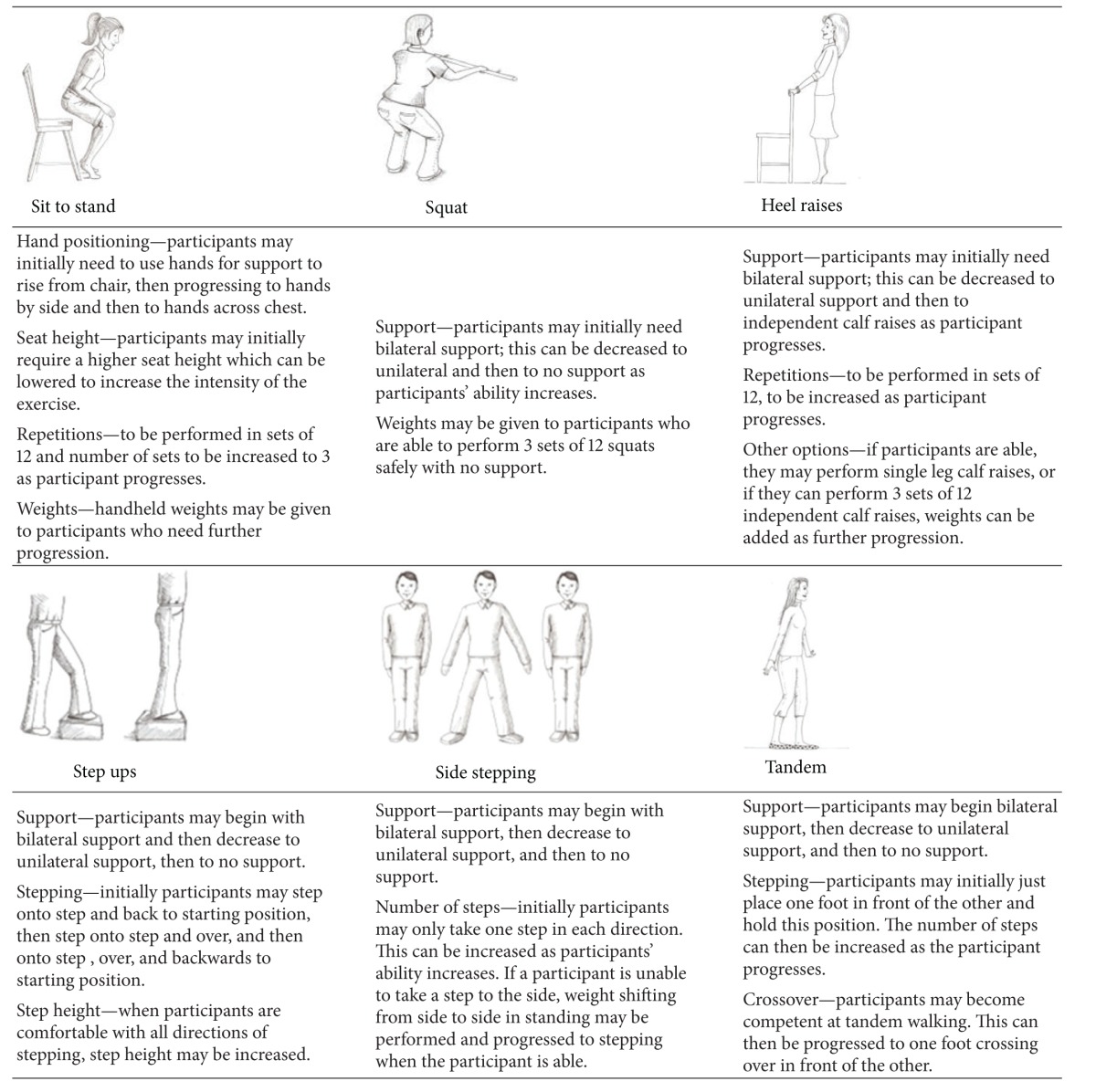

**Table 2:** Baseline comparisons between groups.

	Group physiotherapy *n* = 48	Individual physiotherapy *n* = 35	Yoga *n* = 13	Control *n* = 15	*P* value
Age in years (SIQR)	57 (10)	52 (11)	58 (8)	49 (6)	0.029^b^
Gender					
Male/female (*n*)	18/30	15/20	5/8	2/13	0.247^c^
GNDS mobility section score					
3 (%)	28 (58)	21 (60)	9 (69)	5 (33.3)	0.256^c^
4 (%)	17 (35)	13 (37)	4 (30)	10 (66.7)	
Type of MS					
RRMS (*n*)	13	7	4	5	0.152^c^
SPMS (*n*)	20	16	5	5	
PPMS (*n*)	8	11	2	5	
Unknown (*n*)	7	1	2	0	
Time since diagnosis (years)	18 (9)	13 (8)	15 (8)	10 (3)	0.002^a^
Time since onset of symptoms (years)	22 (11)	20 (13)	21 (14)	15 (7)	0.233^a^
MSIS-29v2 physical component	50.5 (±9)	53.9 (±11.3)	48 (±10)	55 (±9)	0.107^a^
MSIS-29v2 psychological component	18 (5.5)	18 (5)	15 (3)	16.5 (3.25)	0.293
MFIS	40.7 (±16)	46.7 (±14)	**30.4** (±**17**)	47 (±15)	**0.034** ^ a^
BBS	28.5 (±9)	30 (±11.5)	22 (±13)	18 (±6)	0.391^a^
6MWT (m)	105.5 (56)	89 (63)	66 (57)	79 (49)	0.103^a^

SIQR: semi-interquartile range, GNDS: Guys Neurological Disability Scale, RR: relapsing remitting, SP: secondary progressive, PP: primary progressive, MSIS: multiple sclerosis impact scale, MFIS: modified fatigue impact scale, BBS: berg balance scale, 6MWT: six-minute walk test, a = one way ANOVA, b = Kruskal Wallis test, c = Chi Squared test.

**Table 3:** Descriptive statistics for study outcome measures.

	Group physiotherapy *n* = 48	Individual physiotherapy *n* = 36	Yoga *n* = 13	Control *n* = 15
MSIS-29v2 physical component				
Premean (SD)	50.5 (9.5)	54 (11.5)	48.3 (10.5)	55.3(9.5)
Postmean (SD)	45.9 (10.5)	49.4 (12)	49.6 (11.6)	50.5 (11.3)
Mean Difference	−4.54	−4.52	1.3	−4.8
(95% CI)	(−7.5, −1.5)	(−7.9, −1.1)	(−4.7, 7.3)	(−10.4, −0.6)
*P* value (within group)^a^	0.004*	0.012*	0.645	0.08
MSIS-29v2 psychological component				
Premedian (SIQR)	18 (5.5)	18 (5.38)	14 (2.2)	17 (4)
Postmedian (SIQR)	15 (5.7)	17 (4.8)	15 (4)	15 (4.5)
Median difference	−3	−1	1	2
*P* value (within group)^b^	0.005*	0.057	0.281	0.507
MFIS (total score)				
Premean (SD)	40.7 (16.2)	46.6 (14.8)	30.4 (17.1)	49 (15.5)
Postmean (SD)	35.6 (15.6)	39.5 (13.7)	32.5 (19.5)	42.6 (17.1)
Mean difference	−5.1	−7.4	2.15	−6.4
(95% CI)	( −9.1, −1.2)	(−11.6, −3.2)	(−2.9, 7.2)	(−13.1, 0.4)
*P* value (within group)^a^	0.011*	0.001*	0.374	0.062
BBS				
Premean (SD)	28.8 (9.5)	30.4 (11.6)	22.6 (12.6)	24.9 (11.6)
Postmean (SD)	34.5 (9.8)	34.2 (9.8)	27.9 (11.5)	21.8 (11.9)
Mean difference	5.7	3.7	5.3	−3.1
(95% CI)	(−3.6, 7.8)	(−1, 6.3)	(−3.1, 7.5)	(−2.8, 9.0)
*P* value (within group)^a^	<0.0001*	0.008*	<0.0001*	0.258
6-minute walk test (m)				
Premedian (SIQR)	101 (39.5)	83.8 (39.8)	70 (30)	83.5 (44)
Postmedian (SIQR)	121.2 (47.4)	100 (55)	45 (54.5)	90 (35)
Median difference	20.2	16.2	−25	6.5
*P* value (within group)^b^	0.08	0.002*	0.553	0.363

SD: standard deviation, SIQR: semi-interquartile range, MSIS: multiple sclerosis impact scale, MFIS: modified fatigue impact scale, BBS: berg balance scale, *statistically significant at *α* = 0.05, ^a^paired samples *t*-test, ^b^Wilcoxon signed rank test.
